# Inhibition of cancer cell-derived exosomal microRNA-183 suppresses cell growth and metastasis in prostate cancer by upregulating TPM1

**DOI:** 10.1186/s12935-020-01686-x

**Published:** 2021-03-02

**Authors:** Yanping Dai, Xiaoqin Gao

**Affiliations:** 1 Department of Pathology and Pathophysiology, College of Basic Medical Science, Guizhou Medical University, Guiyang, 550004 People’s Republic of China; 2 Center of Reproductive Medicine, Yueyang Maternity and Child Health Hospital, Yueyang, 414000 People’s Republic of China; 3 Zunyi Medical and Pharmaceutical College, No. 2, North Section of Ping an Avenue, Xinpu New District, Zunyi, 563000 Guizhou People’s Republic of China; 4 Centre for Reproductive Research, National School of Medicine Guiyang Medical University Magic, Guiyang, 550004 China

**Keywords:** Prostate cancer, Exosomes, MicroRNA-183, Tropomyosin-1, PC3 cells, LNCaP cells

## Abstract

**Background:**

Emerging evidence continues to highlight the significant role of microRNAs (miRNAs) in the regulation of cancer growth and metastasis. Herein, the current study aimed to elucidate the role of exosomal miR-183 in prostate cancer development.

**Methods:**

Initially, public microarray-based gene expression profiling of prostate cancer was employed to identify differentially expressed miRNAs. The putative target gene TPM1 of miR-183 was subsequently predicted, followed by the application of a luciferase reporter assay and examination of the expression patterns in prostate cancer patients and cell lines. The effects of miR-183 and TPM1 on processes such as cell proliferation, invasion and migration were evaluated using in vitro gain- and loss-of-function experiments. The effect of PC3 cells-derived exosomal miR-183 was validated in LNCaP cells. In vivo experiments were also performed to examine the effect of miR-183 on prostate tumor growth.

**Results:**

High expression of miR-183 accompanied with low expression of TPM1 was detected in prostate cancer. Our data indicated that miR-183 could target and downregulate TPM1, with the overexpression of miR-183 and exosomal miR-183 found to promote cell proliferation, migration, and invasion in prostate cancer. Furthermore, the tumor-promoting effect of exosome-mediated delivery of miR-183 was subsequently confirmed in a tumor xenograft model.

**Conclusions:**

Taken together, the key findings of our study demonstrate that prostate cancer cell-derived exosomal miR-183 enhance prostate cancer cell proliferation, invasion and migration via the downregulation of TPM1, highlighting a promising therapeutic target against prostate cancer.

## Background

Prostate cancer remains the most prevalent cancer among males as well as a leading cause of cancer related death in countries worldwide [1]. The greater majority of prostate cancer cases remain dormant for long periods of time, but metastatic progression can rapidly worsen patient prognosis and lead to death [2]. Moreover, prostate cancer has been well documented to display high avidity for bone metastasis, which represents a central contributing factor of morbidity and mortality in advanced prostate cancer [3]. Metastatic prostate cancer remains largely incurable in spite of the recent identification of novel drug therapy, highlighting the urgent need to develop alternative treatments [4]. Accumulating evidence has been presented emphasizing the correlation between prostate cancer metastasis and microRNAs (miRNAs) [4–6]. Moreover, the dysregulation of exosomal miRNAs has been implicated in metastatic prostate cancer [7]. Thus, exosomal miRNAs represent promising potential biomarkers for the development of metastatic treatment for prostate cancer.

It is widely accepted that exosomes secreted by tumor cells emerge as a promising biomarker for tumor diagnosis on account of their unique composition and function [8]. Crucially, a significant amount of research attention has been recently drawn to exosomal miRNAs owing to their potential as biomarkers for cancer investigation including exosomal miR-129 and miR-375 in castration-resistant prostate cancer [9]. miRNAs represent a class of small, endogenous non-coding RNA that have been shown to negatively regulate gene expression by means of suppressing translation and facilitating the degradation of target mRNA [10]. miRNAs have been reported to possess the ability to influence a wide variety of biological pathways [11]. miRNAs have been reported to play a role in various cellular processes such as cell-cycle regulation, differentiation, apoptosis, and migration, whose alteration has been implicated in the development and progression of various cancers [12]. Findings obtained from a study suggests an under-expressed miRNA, microRNA-183 (miR-183) contributes to the regulation of zinc homeostasis whose decrease was a feature of prostate cancer [13]. Therefore, it might be possible that miR-183 may ultimately contribute to pathogenesis of prostate cancer.

Moreover, miR-183 has been reported to be differentially expressed and capable of targeting TPM1 in prostate cancer was verified in our study. Existing literature has provided evidence verifying the role of miR-183 as an oncogenic role in esophageal squamous cell carcinoma by targeting PDCD4 [14]. Furthermore, Tropomyosin-1 (TPM1) has been demonstrated to function as a tumor suppressor gene in tongue squamous cell carcinomas [15]. Hence, we asserted the notion that miR-183 contributes to the metastatic progression of prostate cancer cells by targeting TPM1. Based on the exploration of previous studies, we hypothesized that the transfer of miR-183 *via* prostate cancer-derived exosomes could enhance cell proliferation invasion and migration in prostate cancer, with the objective of identifying a novel therapeutic strategy for the treatment of prostate cancer.

## Materials and methods

### Ethics statement

The current study was conducted with the approval of the Ethics Committee of Guizhou Medical University. All experiments involving animals were performed in strict adherence with the recommendations in the Guide for the Care and Use of Laboratory Animals of the National Institutes of Health.

### Datasets and bioinformatics analysis

Prostate cancer-associated miRNA (GSE14857 and GSE64318) and mRNA (GSE30994) expression profiles were retrieved in the Gene Expression Omnibus (GEO) database (https://www.ncbi.nlm.nih.gov/geo). Differentially expressed miRNA and mRNA were analyzed using the limma package and identified using |log2 fold change (FC)| > 2.0 and p value < 0.05 as the threshold. Heatmaps were plotted using heatmap package in R language. The Venn online analysis tool (Calculate and draw custom Venn diagrams) (http://bioinformatics.psb.ugent.be/webtools/Venn/) was employed to construct a Venn map of differential miRNAs from two miRNA datasets followed by an analysis of the intersecting region. The target gene candidates for miR-183 were predicted using the database including miRDB (http://www.mirdb.org/), miRDIP (http://ophid.utoronto.ca/mirDIP/) and miRWalk (http://mirwalk.umm.uni-heidelberg.de/). The intersection between the predicted target genes and the top 100 most significantly downregulated mRNAs from the GSE30994 dataset was obtained. The expression of TPM1 in prostate cancer based on The Cancer Genome Atlas (TCGA) and in normal samples was analyzed by GEPIA (Gene Expression Profiling Interactive Analysis) (http://gepia.cancer-pku.cn/index.html).

### Cell culture and transfection

Normal prostate epithelial cell line RWPE-1(ATCC^®^ CRL-11609™), androgen-dependent (LNCaP) and androgen-independent (PC3) human prostate cancer cell lines (ATCC^®^ CRL-1740™) were obtained from ATCC (Manassas, VA, USA). The cell lines were cultured with modified Dulbecco’s Modified Eagle Medium (DMEM; Invitrogen, Carlsbad, CA, USA) supplemented with 10% fetal bovine serum (FBS; Gibco, Carlsbad, CA, USA) and 100 U/mL penicillin and streptomycin at 37 °C with 5% CO_2_. The cells were subcultured upon reaching 80–90% confluence. The cells exhibiting logarithmic growth were subsequently seeded into a 6-well plate at 4 × 10^5^ cells per well. LNCaP and PC3 cells were prepared for transfection as per the instruction of the Lipofectamin2000 kit (11668-019, Invitrogen, Carlsbad, CA, USA). Upon reaching 80% confluence, the LNCaP cells were transfected with miR-183 mimic, miR-183 inhibitor, small interfering RNA targeting TPM1 (si-TPM1), TPM1 overexpression plasmid (oe-TPM1) or their negative controls (mimic-NC, inhibitor-NC, si-NC and oe-NC). The PC3 cells were transfected with miR-183 mimic, miR-183 inhibitor, mimic-NC or inhibitor-NC. All aforementioned transfection plasmids were purchased from Shanghai GenePharma Co., Ltd (GenePharma, Shanghai, China).

### Dual-luciferase reporter gene assay

The TPM1 3′-untranslated region was identified to contain the miR-183 predicted targeted binding sites. Dual-luciferase reporter gene assay was subsequently performed to determine whether TPM1 is a direct target of miR-183. TPM1 3′-untranslated region (3′UTR) was artificially synthesized and inserted into psiCHECK-2 vector (Promega, Madison, WI, USA) *via* SpeI and Hind III sites. Site-directed, mutagenesis, complementary sequence was performed on the basis of the TPM1 wild type (WT) sequence. The target segment was inserted into psiCHECK-2 plasmid *via* T4 DNA ligase, followed by restriction enzyme digestion. Two recombinant plasmids namely, TPM1-WT, and TPM1 mutant (TPM1-MUT) were co-transfected into cells with miR-183 mimic and the negative control (NC) of miR-183, respectively. The cells were then lysed following a 48 h period of transfection. Luciferase activity was measured using the Luminometer TD-20/20 (Promega, Madison, WI, USA) using dual-luciferase reporter assay system kit (Promega, Madison, WI, USA).

**Table 1:** Primer sequences for RT-qPCR

Gene	Primer sequence (5′-3′)
miR-183	Forward: 5′-CGCGGTATGGCACTGGTAGA-3′
	Reverse: 5′-AGTGCAGGGTCCGAGGTATTC-3′
TPM1	Forward: 5′-GTGGGGAAAACACATACAAAAAG-3′
	Reverse: 5′-CTTCCTGTTGACTCTATCATTGG-3′
GAPDH	Forward: 5′-CCTGGCCAAGGTCATCCATG-3′
	Reverse: 5′-GGAAGGCCATGCCAGTGAGC-3′
U6	Forward: 5′-CTCGCTTCGGCAGCACA-3′
	Reverse: 5′-AACGCTTCACGAATTTGCGT-3′

*RT-qPCR* reverse transcription quantitative polymerase chain reaction, *miR-183* microRNA-183, *TPM1* tropomyosin-1, *GAPDH* glyceraldehyde-3-phosphate dehydrogenase, *F* forward, *R* reverse

### Cell counting kit-8 (CCK-8) assay

LNCAP and PC3 cell suspension were seeded into a 96-well plate at a density of 100 µL/well with the cell culture plate placed into an incubator at 37 ℃ with 5% CO_2_. CCK-8 solution was added to each well following 1–4 h culture. The control group was set by adding medium only. After 48 h, 20 µL of 5 mg/mL CCK-8 solution was added for an additional 4 h culture. In the presence of an electron-coupled reagent, water-soluble tetrazolium salt-8 (WST-8) is reduced to orange-yellow formazan by dehydrogenase in the mitochondria. A darker hue was considered to be indicative of faster rate of cell proliferation. The absorbance (A) was determined at a wavelength of 450 nm using an automatic microplate reader.

### Transwell assay

Transwell assay was employed to evaluate cell migration. Plasmid DNA was transfected into cells for 24 h, after which the cells were digested for detachment purposes, with the single cell suspension diluted to a concentration of 1 × 10^6^ cells/mL by culture medium containing 1% FBS. Complete medium containing 10% FBS was subsequently added to the bottom of a 24-well plate at 600 µL per well. The Transwell chamber was placed on the 24-well plate after which 100 µL of cell suspension was added into the chamber and cultured at 37 ℃. After 24 h, the Transwell chamber was removed. The culture medium with cells that failed to exhibit any migration inside the chamber was removed using a cotton swab. The Transwell chamber was washed twice with phosphate buffered saline (PBS), fixed with formaldehyde and acetic acid (3:1) for 15–30 min, and then stained with 1% gentian violet for 15 min. The migrated cells were tallied under a microscope (TE2000, Nikon, China).

Cell invasion was assessed using a Matrigel coated Transwell chamber (356234, Corning Incorporated, NY, USA) diluted with pre-cooled serum-free DMEM. 100 µL of Matrigel (356234; Corning, USA) was added to the Transwell chamber. The Transwell chamber was then placed into a 24-well plate and incubated at 37 ℃ for at least 4–5 h. The basement membrane was hydrated after which the Matrigel was washed using a serum-free medium. The cells were treated with trypsin and washed for 3 times with the medium. The cells were subsequently resuspended to a final density of 1 × 10^5^ cells/mL in medium containing with 1% FBS. A total of 600 µL medium containing 5 µg/mL fibronectin was added to the basolateral chamber and 100 µL of cell suspension was added to the apical chamber. The cells were then cultured for 20–24 h at 37 ℃. The cells failing to migrate from the chamber were removed using a cotton swab. The membrane was removed and stained with 500 µL of 1% crystal violet. The membrane was immersed in the medium for 30 min at 37 ℃. Four replicate wells were set in each group, which were repeated 3 times. The number of stained cells was counted under a microscope. Four fields of view were selected under a microscope. The stained cells were finally counted and recorded.

### Isolation and identification of prostate cancer cells-derived exosomes

PC3 cells were cultured in a 15 cm culture dish. After reaching 80% confluence, the cells were washed 3 times with 20 mL PBS and incubated with exosome-free culture medium (10% exosome-depleted FBS + RMPI1640) for 2 days. The cell medium was then collected and centrifuged at 3000×*g* for 15 min at 4 °C. The supernatant was concentrated via centrifugation at 5000*g* at 4 ℃ for 50 min. The concentrate was added to ExoQuick exosome extract at a 1:1 ratio overnight at 4 °C. The following day, the mixture was centrifuged at 1500*g* for 30 min at 4 °C. After removal of the supernatant, the precipitate was collected and washed using sterile PBS, and then stored at − 80 °C.

The morphology of the exosomes was assessed by transmission electron microscopy and the particle size of the exosomes was measured by a dynamic light scattering (DLS) (Mastersizer 3000, Malvern Panalytical). Western blot analyses were developed to determine the extracted exosome and the expression of the protein markers HSP70 (1:1000, ab2787, abcam, Cambridge, UK) and CD63 (1:1000, ab216130, abcam, Cambridge, UK), as well as calnexin protein that was negatively expressed in exosomes but positively expressed in cells in the supernatant after exosome isolation.

The content of the exosome surface marker CD63 was detected by flow cytometry. Briefly, the exosomes were isolated from PC3 were resuspended in 1 mL PBS containing 1% BSA and incubated at room temperature for 30 min in order to block non-specific antigen. Centrifugation was performed at 1000 rpm for 5 min, after which the supernatant was discarded, with the exosomes subsequently resuspended with 200 L/EP tube PBS. CD63-PE antibody was added to each exosome and incubated at room temperature for 30 min. The samples without any antibody added were employed as the blank control and anti-human IgG labeled by PE as homotype control. After centrifugation at 1000 rpm for 5 min, the exosomes were resuspended with 1 mL PBS containing 1% BSA followed by flow cytometry analysis using Guava easyCyte™ system.

### Co-culture of PC3 cells-derived exosomes with LNCAP cells

In order to inhibit exosomal release from the PC3 cells, PC3 cells were treated with the exosome inhibitor GW4869 with PBS treatment as control. PC3 cells were plated in a 6-well plate at 1 × 10^6^ cells per well. Upon confluence reaching 80–90%, cells were treated with 10% of GW4869 (D1692-5MG, Sigma-Aldrich, St. Louis, MO, USA), and the control group was treated with 0.005% dimethyl sulphoxide (DMSO). Both the cells and the supernatant were collected after 24 h of treatment.

The PC3 cells were transfected with mimic-NC, miR-183-mimic, inhibitor-NC, or miR-183-inhibitor. The LNCAP cells were transfected with si-TPM1 or si-NC and subsequently seeded into a 24-well plate. When the cell confluence had reached approximately 60%, the transfected LNCAP cells co-cultured with the exosomes derived from the transfected PC3 cells for 48 h.

The CFSE dye (ab113853, abcam, Cambridge, UK) was added in a 1:1000 dilution to 20 µg of the exosomal suspension derived from the transfected PC3 cells and cultured at 37 °C for 15 min. The mixture was then added to PBS, washed and centrifuged at 100,000*g* for 70 min. Finally, CFSE-tagged exosomes were co-cultured with LNCAP cells. The uptake of exosomes by the LNCAP cells was observed under a fluorescence microscope after 24 h of coculture.

### Reverse transcription-quantitative polymerase chain reaction (RT-qPCR)

The total RNA from the tissues was extracted using Trizol reagent (Invitrogen, Carlsbad, CA, USA). Reverse transcription was performed according to the instructions of TaqMan™ MicroRNA Reverse Transcription Kit (4366596) and High-Capacity cDNA Reverse Transcription Kit (4368813) both from Thermo Fisher Scientific (Waltham, MA, USA) in order to generate cDNA. Real-Time Quantitative fluorogenic PCR assay was developed in accordance with the instructions of the SYBR^®^Premi × Ex TaqTM (Tli RNaseH Plus) kit (RR820A, TaKaRa, Japan) using PCR instrument (ABI7500, Thermo, Waltham, MA, USA). The primers for miR-183 and TPM1 were synthesized by Sangon Biotech Co. Ltd (Shanghai, China). The relative expression of the target genes was measured using the 2^−ΔΔCt^ method normalized to GAPDH or U6 (Invitrogen, Carlsbad, CA, USA) mRNA levels. Sequences for primers are listed in Table 1.

### Western blot analysis

Total protein from tissues and cells was extracted using radio-immunoprecipitation assay (RIPA) kit (Beijing Solarbio Science and Technology Co., Ltd, Beijing, China). Protein concentration was determined using a BCA protein assay kit (GBCBIO Technologies Inc., Guangzhou, China). The proteins were subsequently separated using 10% sodium dodecyl sulfate-polyacrylamide gel electrophoresis (SDS-PAGE). Following separation, the proteins were transferred onto a polyvinylidene fluoride (PVDF) membrane (Merck Millipore, Billerica, MA, USA), and subsequently blocked for 2 h with Tris Buffered Saline with Tween (TBS/T) containing 5% skimmed milk. The PVDF membranes were incubated with primary antibody β-Tubulin (ab210797, 1:1000, Rabbit), TPM1 (ab38898, 1: 1000, Rabbit), CD63 (ab216130, 1:1000, Rabbit), HSP70 (ab181606, 1:1000, Rabbit), and Calnexin (ab22595, 1:1000, Rabbit) overnight at 4 °C. The membrane was then incubated with the secondary antibody goat anti-rabbit immunoglobulin G (IgG) (ab150077, 1:1000, Abcam, UK) at room temperature. Enhanced chemiluminescence (ECL) was applied for visualization. The image was subjected to gray scale analysis using Image J software.

### Tumor xenografts in nude mice

Male athymic NCr-nu/nu mice (aged 6 weeks) were provided by the Experimental Animal Centre of Wuhan University and raised in laminar flow cabinets under specific pathogen-free (SPF) conditions. Indoor UV irradiation was performed on a regular basis. The housing environment was maintained with room temperature at 24–26 °C and 40–60% relative humidity. The mice were classified into two groups (n = 6): exo-inhibitor-NC group (injection of LNCaP + PC3 exo-inhibitor-NC) and exo-miR-183-inhibitor group (injection of LNCaP + PC3 exo-miR-183-inhibitor). Each mouse was then administered with a subcutaneous injection of 1 × 10^6^ LNCaP cells. Moreover, mice were injected with PBS, or exosomes derived from the PC3 cells transfected with inhibitor-NC or miR-183-inhibitor *via* the tail vein 5 days per week for 30 days. Tumor growth indicators were recorded daily [16]. On the 30th day, the nude mice were anesthetized and euthanized using CO_2_ and 50% O_2_ mixed gas. The volume and weight of tumor were measured.

### Terminal deoxynucleotidyl transferase (TDT)-mediated dUTP-biotin nick end-labeling (TUNEL)

Cell apoptosis was assessed based on the instructions of the TUNEL Kit (Beyotime Institute of Biotechnology, Jiangsu, China). The tissue sections were then dewaxed with xylene, gradient alcohol rehydrated, and immersed in 3% hydrogen peroxide (H_2_O_2)_ solution at room temperature for 10 min. After the sections had been washed with PBS, the tissue proteins were removed following the addition of 50 µL of 20 µg/mL Proteinase K for 20 min at room temperature. After three PBS washes, the tissues were added citrate buffer for 30 min for antigen retrieval. The tissues were incubated with 50 µL of TdT solution for 1 h under conditions void of light. The reactions without TdT were employed as the negative control. The tissues were incubated with 50 µL peroxidase-labeled-anti-digoxigenin at 37 °C for 30 min under dark conditions, followed by three PBS washes. DAB (ZSGB-BIO, China) was used to develop for 10 min. Following washing with PBS, the tissues were counterstained with hematoxylin and observed under a microscope (Nikon, Japan).

### Statistical analysis

All experimental data were analyzed using SPSS 21.0 statistical software (IBM SPSS Statistics, Chicago, IL, USA). Measurement data were expressed as the mean ± standard deviation. Unpaired data obeying normal distribution and homogeneity between two groups were compared using an unpaired *t*-test. Comparisons among multiple groups were performed by one-way analysis of variance (ANOVA) with the application of a Tukey’s post hoc test. Time-based measurements within each group was analyzed using repeated measurement ANOVA, followed by a Bonferroni’s post-hoc test. A value of *p* < 0.05 was considered to be indicative of statistically significant difference.

## Results

### miR-183 is upregulated in prostate cancer

**Fig. 1:**
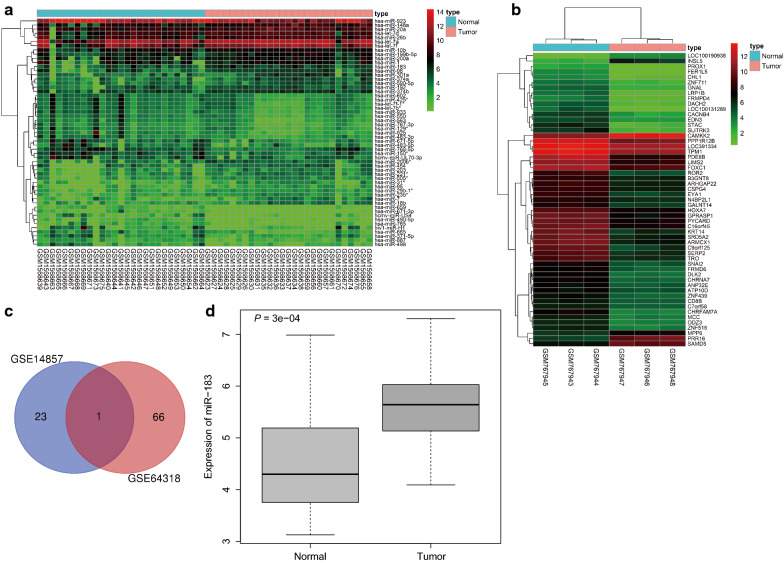
The differentially expressed miRNAs in GSE14857 and GSE64318 profiles. a Heat map of differentially expressed miRNAs in prostate cancer in GSE64318 profile, the X axis indicates the sample number, the Y axis indicates the miRNA name. The upper bar indicates the type of sample, the blue refers to normal sample, and the red refers to tumor sample. The histogram at the upper right indicates the color gradation, red refers to high expression, and green refers to low expression. The left tree indicates miRNA expression clustering, each rectangle in the figure refers to one sample expression value of a miRNA. **b** Heat map of differentially expressed genes in prostate cancer among GSE30994 profile, the X axis indicates the sample number, the Y axis indicates the gene name. The upper bar indicates the type of sample, the blue refers to normal sample, and the red refers to tumor sample. The histogram at the upper right indicates the color gradation, red refers to high expression, and green refers to low expression. The upper tree indicates sample expression clustering, the left tree indicates gene expression clustering, each rectangle in the figure refers to one sample expression value of a gene. a PPI network of DEGs in bladder cancer. **c** Venn analysis of differentially expressed miRNAs in GSE14857 and GSE64318 profiles, blue indicates differentially expressed miRNAs in GSE14857 profile, red indicates differentially expressed miRNAs in GSE64318 profile, the middle part indicates the intersection of the two profiles, and the figure indicates the number of differentially expressed miRNAs. **d** High expression of miR-183 in prostate cancer, X axis indicates sample type, Y axis indicates normal sample, and tumor indicates prostate cancer sample

The dataset GSE14857 of miRNA expression in tumor tissue and normal adjacent tissue with untreated prostate carcinoma, the dataset GSE64318 of miRNA expression in prostate cancer-derived tumor and adjacent normal tissues and the dataset GSE30994 of prostate cancer-specific gene expression in tissue of patients with prostatic hyperplasia were retrieved from the GEO database. There were 8 miRNAs that were significantly upregulated (*p* < 0.05) in addition to 16 miRNAs significantly downregulated (*p* < 0.05) in prostate cancer among GSE14857; 38 miRNAs significantly upregulated (*p* < 0.05) and 29 miRNAs significantly downregulated (*p* < 0.05) in GSE64318; 1223 mRNA significantly upregulated (*p* < 0.05) and 1777 mRNA significantly down-regulated (*p* < 0.05) in GSE30994. An expression heat map was constructed to display the top 55 miRNAs or mRNAs among the GSE64318 and GSE30994 profiles (Fig. 1a, b). In order to further screen the prostate cancer-associated miRNAs, all differential miRNAs in the GSE14857 and GSE64318 profiles were selected for Venn analysis (Fig. 1c). The results indicated that only miR-183 was present in both GSE14857 and GSE64318 profiles. Moreover, miR-183 was highly expressed in prostate cancer samples of the two profiles (Fig. 1d). These results were consistent with previous literature [13, 17].

### Overexpression of miR-183 promotes cell proliferation, invasion, and migration in prostate cancer cells

**Fig. 2:**
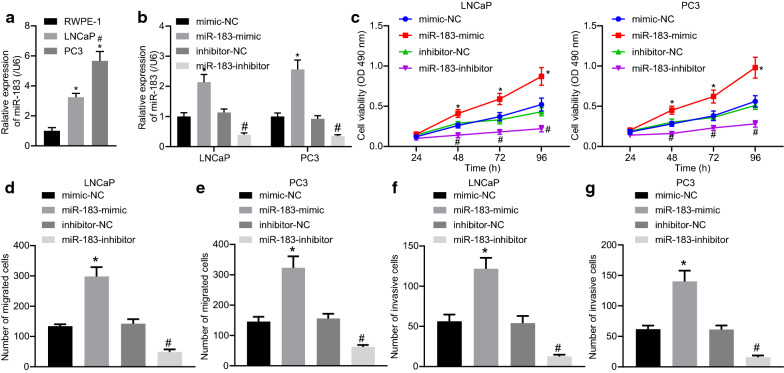
Overexpression of miR-183 promoted cell proliferation and invasion in prostate cancer. **a **Determination of miR-183 expression in prostate cancer by RT-qPCR assay, showing high expression of miR-183 in RWPE-1, LNCAP, and PC3 cells. *p < 0.05 vs. RWPE-1 cells, ^#^*p* < 0.05 vs. LNCAP cells. **b** Determination of miR-183 expression following overexpression and inhibition of miR-183 in LNCAP and PC3 cells by RT-qPCR assay. **c** Cell viability examined by MTT assay. **d**, **e** Transwell assay for the detection of the effect of miR-183 on cell migration, blue indicates the migrated cells. **f**, **g** Transwell assay for the detection of the effect of miR-183 on cell invasion, blue indicates the invaded cells *p < 0.05 vs. mimic-NC group, ^#^*p* < 0.05 vs. inhibitor-NC group. The miR-183-mimic group, miR-183-inhibitor group, mimic-NC group, and inhibitor-NC group referring to LNCaP or PC3 cells respectively transfected with miR-183 mimic, miR-183 inhibitor, mimic-NC, inhibitor-NC. Cell experiments were repeated three times

Next, to explore the functional role of miR-183 in prostate cancer cells, overexpression or inhibition of miR-183 was conducted in two prostate cancer lines LNCaP and PC3 exhibiting higher levels of miR-183 expression relative to the RWPE-1 normal line (*p* < 0.05; Fig. 2a). As expected, miR-183 mimic transfection was accompanied with an increased expression of miR-183 and miR-183 inhibitor transfection resulted in a reduction, as depicted by the RT-qPCR findings (*p* < 0.05; Fig. 2b). Overexpression of miR-183 led to a marked increase in the viability of prostate cancer cells, while the cell viability was significantly reduced by the miR-183 inhibitor (*p* < 0.05; Fig. 2c). Thus, the results obtained demonstrate that miR-183 may play a role in the proliferation of prostate cancer cells.

A Transwell assay was performed to assess the effect of miR-183 on migration and invasion of PC3 and LNCaP cells. The results obtained illustrated that the migration of both PC3 and LNCaP cells was notably elevated following miR-183-mimic transfection while decreased by miR-183-inhibitor transfection (*p* < 0.05; Fig. 2d, e). Similar results were seen in the transwell invasion assay (Fig. 2f, g). Altogether, the data obtained suggested that miR-183 promotes prostate cancer cell migration and invasion.

### miR-183 targets TPM1 in prostate cancer

**Fig. 3:**
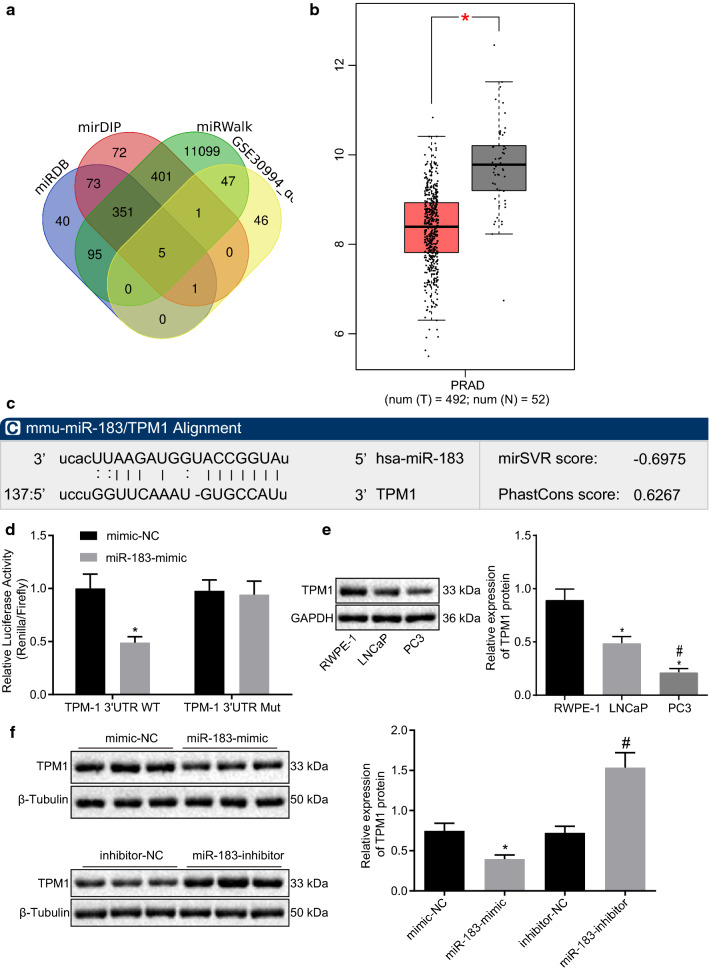
miR-183 targets and negatively regulates the expression of TPM1 in prostate cancer. **a** Prediction of miR-183 downstream target genes, blue, red, and green indicates prediction results of miRDB, mirDIP and miRWalk database respectively, yellow indicates the top 100 significantly downregulated genes in GSE30994 profile, the region of number 5 referring the intersection of 4 data sets. **b** Expression of TPM1 in prostate cancer and normal tissues in GEPIA website. **c** The binding sites of miRNA and TPM1-3′UTR predicted by the software. **d** The interaction between miR-183 and TPM1 assessed by dual-luciferase reporter gene assay. **e** The expression of TPM1 in RWPE-1, LNCaP and PC3 cell lines measured by Western blot analysis. *p < 0.05 vs.. RWPE-1 cells, ^#^*p* < 0.05 vs. LNCAP cells. **f** The expression of TPM1 measured by Western blot analysis. *p < 0.05 vs. mimic-NC, ^#^*p* < 0.05 vs. inhibitor-NC group. The miR-183-mimic group, miR-183-inhibitor group, mimic-NC group, and inhibitor-NC group refer to LNCaP cells respectively transfected with miR-183 mimic, miR-183 inhibitor, mimic-NC, inhibitor-NC, respectively. Cell experiment was repeated three times

To further clarify the miR-183’s downstream gene targets, we predicted target genes of miR-183 using miRDB, mirDIP and miRWalk databases. Venn analysis was conducted to analyze the predicted result as well as the top 100 most significantly downregulated genes in mRNA profile GSE30994 (Fig. 3a). The results indicated that five genes including NTN4, N4BP2L1, FRMD6, CACNB4, and TPM1 were present in the overlapping region across all four sets of data. In contrast to miR183 expression, the expression of TPM1 in prostate cancer was distinctly lower than that in the normal samples from the GEPIA database (Fig. 3b), supporting our earlier in silico prediction.

The biological prediction website indicated the presence of a binding site between the seed sequence of miR-183 and the 3′UTR end of TPM1 (Fig. 3c). To experimentally confirm TPM1 as a direct target of miR-183, we performed dual-luciferase reporter gene assay. The results suggested that in comparison to the LNCaP cells transfected with the mimic-NC, the luciferase activity of the WT-TPM1 3′UTR was significantly decreased in LNCaP cells transfected with the miR-183-mimic (*p* < 0.05); whereas there was no significant difference in luciferase activity of mutant-TPM1 3′UTR in the LNCaP cells transfected with miR-183-mimic (*p* < 0.05; Fig. 3d).

Western blot analysis results demonstrated that the expression of TPM1 in RWPE-1, LNCaP and PC3 cell lines decreased (*p* < 0.05; Fig. 3e). The expression of TPM1 was significantly diminished in the setting of overexpressed miR-183 in LNCaP cells. However, silenced miR-183 led to an increase in TPM1 expression (*p* < 0.05; Fig. 3f). The aforementioned results indicated that TPM1 was poorly expressed in prostate cancer, while miR-183 targeted TPM1 expression in prostate cancer cells, which may contribute to its diminished expression.

### Exosomes derived from PC3 cells can be delivered into prostate cancer cells

**Fig. 4:**
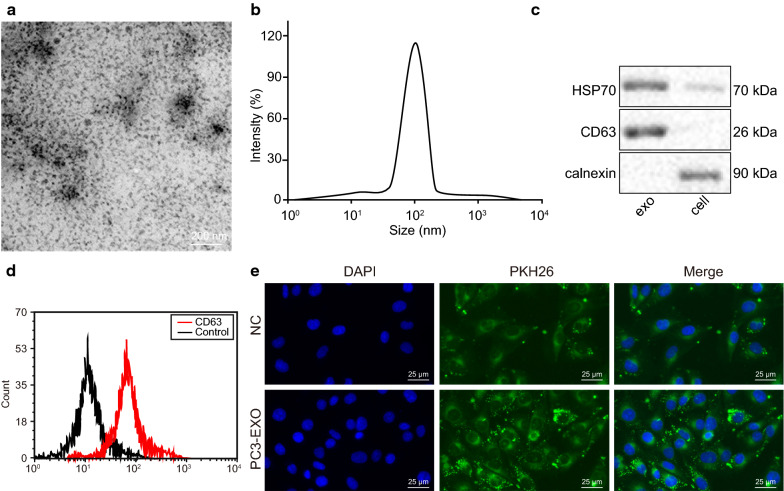
Exosomes derived from PC3 cells can be taken up by LNCaP cells. **a** Exosomes derived from PC3 cells were observed under a transmission electron microscope (× 5000). The size and morphology of vesicles were not uniform. **b** Detection of exosome diameter by DLS. **c** The expression of HSP70 and CD63 measured by Western blot analysis, the content of both proteins in exosomes higher than that in supernatant, lane 1 indicates exosome and lane 2 indicates cell. **d** The content of CD63 examined by flow cytometry. **e** The image of laser confocal microscopy. Exosomes derived from PC3 cells labeled by CFSE (green fluorescence) could be delivered into LNCAaP cells (× 400). Measurement data were expressed as mean ± standard deviation. Comparison between two groups was analyzed by unpaired *t*-test. Each experiment was repeated three times

Existing literature has emphasized cancer cell-derived exosomes as a crucial source of miR-183 (Jian, Wei, Yan, et al.). Thus, we isolated the exosomes from prostate cancer cells. Round or elliptical membranous vesicles with identical morphology were examined under an electron microscope (Fig. 4a). DLS was employed to examine the size of the exosomes with a diameter of 30–120 nm (Fig. 4b). The protein expression of the HSP70 and CD63 were measured by Western blot analysis. The results indicated that the exosomes expressed CD63 and HSP70, but not Calnexin (Fig. 4c). Flow cytometry findings indicated that the expression of exosome surface marker CD63 was significantly higher in isolated exosomes versus control (*p* < 0.05; Fig. 4d).

In order to ascertain whether the exosomes derived from PC3 cells are taken up by LNCaP cells is examined in our study. PC3 cell-derived exosomes labeled by CFSE (green) were incubated with LNCaP cells for 24 h and observed under a laser confocal microscopy. Our findings indicated that green signal-labeled vesicles were observed to have adhered to the LNCaP cell membrane and fusing inside the cell membrane (Fig. 4e), suggesting uptake of PC3 cells derived exosomes by LNCaP cells.

### PC3 cell-derived exosomes carry miR-183 that downregulates TPM1

**Fig. 5:**
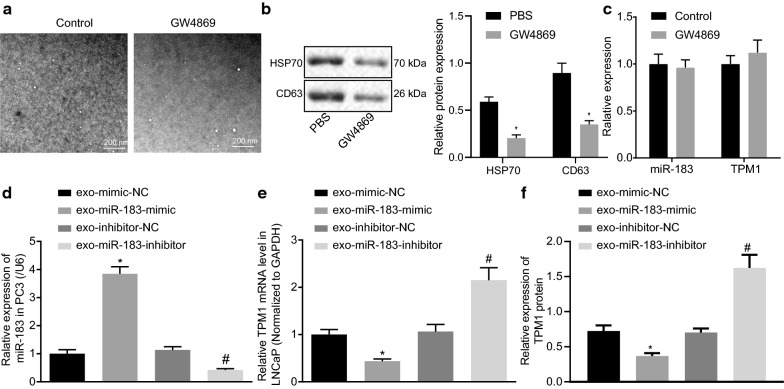
PC3 cells-derived exosomal miR-183 downregulates TPM1. **a** The secretion of exosomes observed under an electron microscope (× 5000). **b** Quantification of HSP70 and CD63 in exosomes detected by Western blot. **c** The expression of f miR-183 and TPM1 in PC3 cells detected by RT-qPCR assay. **d** The expression of f miR-183 analyzed by RT-qPCR assay. **e** The expression of TPM1 in LNCaP cells evaluated by RT-qPCR assay. **f** The expression of TPM1 in LNCaP cells evaluated by Western blot assay. *p < 0.05 vs. exo-mimic-NC group, ^#^*p* < 0.05 vs. exo-inhibitor-NC group. The exo-mimic-NC group, exo-miR-183-mimic group, exo-inhibitor-NC group, and miR-183-inhibitor group refer to exosome derived from PC3 cells respectively transfected with mimic-NC, exo-miR-183-mimic, inhibitor-NC and miR-183-inhibitor. The exosomes derived from the transfected PC3 cells were co-cultured with LNCAP cells Measurement data were expressed as mean ± standard deviation. Comparison between two groups was analyzed using independent sample *t*-test. Cell experiment was repeated three times

After the PC3 cells were treated with GW4869 for 24 h, the secreted exosomes were observed under an electron microscope. The results highlighted an inhibition of exosome secretion in the PC3 cells after GW4869 treatment (Fig. 5a). The exosomes were collected, and the contents of HSP70 and CD63 in the exosomes were detected by Western blot analysis to quantify the exosomes. The results demonstrated that the secretion of exosomes in PC3 cells was notably reduced following the introduction of the exosome inhibitors (Fig. 5b). The expression of miR-183 and TPM1 in PC3 cells was evaluated by RT-qPCR assay. The results demonstrated that blocking exosome secretion did not affect the expression of miR-183 and TPM1 in PC3 cells (Fig. 5c).

Next, exosomes derived from PC3 cells by miR-183-mimic or miR-183-inhibitor transfection were extracted and evaluated by RT-qPCR assay. The results demonstrated that the expression of miR-183 in the exosomes derived from miR-183-mimic-transfected PC3 cells was notably higher than that in mimic-NC. The expression of miR-183 was significantly lower in exosomes derived from miR-183-inhibitor transfected PC3 cells compared with exosomes derived from PC3 cells transfected by inhibitor-NC (*p* < 0.05; Fig. 5d).

We subsequently set out to further investigate whether the PC3 cells-derived exosomal miR-183 affects the expression of TPM1 in LNCaP cells. Following co-incubation of exosomes derived from differently treated PC3 cells with LNCaP cells, the expression of TPM1 in LNCaP cells was assessed by RT-qPCR and Western blot assay. The results demonstrated that the expression of TPM1 was significantly decreased in LNCaP cells co-cultured with miR-183-mimic-transfected PC3 cells-derived exosomes compared with those co-cultured with exosomes derived from PC3 cells by miR-183-NC transfection. The expression of TPM1 was significantly increased in LNCaP cells co-cultured with exosomes derived from miR-183-inhibitor transfected PC3 cells compared with those co-cultured with exosomes derived from miR-183-NC transfected PC3 cells (Fig. 5e, f). In conclusion, PC3 cells-derived exosomal miR-183 negatively regulates the expression of TPM1 in LNCaP cells.

### Inhibition of prostate cancer exosomes carrying miR-183 inhibits prostate cancer development by promoting TPM1 expression

**Fig. 6:**
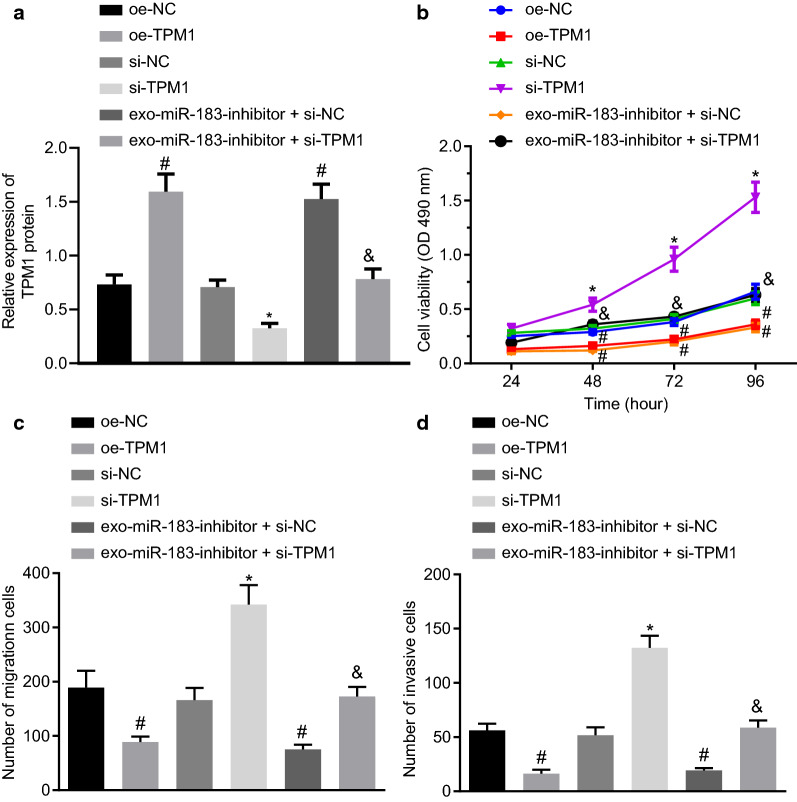
PC3 cells-derived exosomal miR-183 promotes the development of prostate cancer through downregulation of TPM1. **a** Expression of TPM1 in LNCaP cells detected by Western blot analysis. **b** Detection for proliferation in LNCaP cells by CCK-8 assay. **c**, **d** Detection for migration and invasion in LNCaP cells by Transwell assay (× 200). *p < 0.05 vs. si-NC group, ^#^*p* < 0.05 vs. oe-NC group. The oe-TPM1 group, si-TPM1 group, oe-NC group, and si-NC group refer to LNCaP cells transfected with oe-TPM1, si-TPM1 and their negative controls. The exo-miR-183-inhibitor + si-TPM1 group and exo-miR-183-inhibitor + si-NC group refer to si-TPM1 transfected-LNCaP cells co-cultured with exosome derived from PC3 cells transfected with miR-183-inhibitor and si-TPM1, The exo-miR-183-inhibitor + si-NC group refers to si-NC transfected-LNCaP cells co-cultured with exosome derived from PC3 cells transfected with miR-183-inhibitor. Measurement data were expressed as mean ± standard deviation. Comparisons among multiple groups were conducted by one-way analysis of variance (ANOVA) with Tukey’s post hoc test. Statistical analysis in relation to time-based measurements within each group was realized using repeated measurement ANOVA, followed by a Bonferroni’s post-hoc test. n = 6

The change of TPM1 expression in LNCaP cells was detected by Western blot analysis. Following the overexpression (oe) or knockdown (si) of TPM1 in cells and treatment with exosomes, the results revealed that TPM1 expression was significantly increased by oe-TPM1 or exo-miR-183-inhibitor but was reduced by si-TPM1. In the presence of exo-miR-183-inhibitor, si-TPM1 caused decline of TPM1 expression (Fig. 6a). Thus, exosomes derived from PC3 cells carrying miR-183 regulates the expression of TPM1 in LNCaP cells.

CCK-8 and Transwell assay were performed in order to assess the proliferation, migration, and invasion of the LNCaP cells. The results demonstrated that cell viability was decreased by oe-TPM1 or exo-miR-183-inhibitor. However, cell viability was augmented by si-TPM1, which was annulled by further treatment with exo-miR-183-inhibitor (*p* < 0.05; Fig. 6b). Similar results were obtained in relation to the migration and invasion assay findings whereby the silencing of TPM1 abolished the exosome mediated reduction in migration and invasion (*p* < 0.05; Fig. 6c, d). Altogether, a lower expression of TPM1 was found to play a stimulatory role on proliferation, migration, and invasion of prostate cancer cells; co-incubation with exosomes decreased the expression of miR-183 and increased the expression of TPM1, thereby inhibiting the proliferation, migration and invasion in LNCaP cells.

### **Inhibition of PC3 cells-derived exosomal miR-183 diminished prostate tumorigenesis*in vivo***

**Fig. 7:**
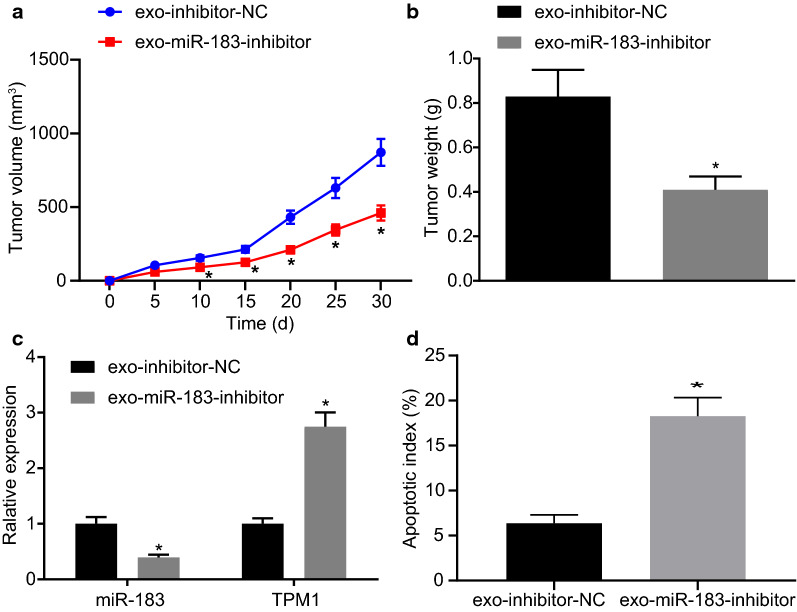
Inhibition of the expression of exosomal miR-183 impedes the development of prostate tumors. **a** Volume changes of tumor growth (n = 6). **b** Weight change of tumor growth (n = 6). **c** The expression of miR-183 and TPM1 in tumor tissues of nude mice detected by RT-qPCR assay. **d** Apoptosis in tumor tissues of nude mice detected by TUNEL assay (× 200). *p < 0.05 vs. exo-inhibitor-NC. exo-inhibitor-NC refers to mice injected with exosomes derived from inhibitor-NC transfected-LNCaP cells. exo-miR-183-inhibitor refers to mice injected with exosomes derived from miR-183-inhibitor transfected-LNCaP cells. Measurement data were expressed as mean ± standard deviation. Statistical analysis in relation to time-based measurements within each group was realized using repeated measurement ANOVA, followed by a Bonferroni’s post-hoc test. Each experiment was repeated three times

We set out to further investigate the effect of PC3 cells-derived exosomal miR-183 on subcutaneous tumor growth in nude mice. The tumor volume (Fig. 7a) and final tumor weights (Fig. 7b) of mice injected with exosomes carrying miR-183-inhibitor were markedly decreased (*p* < 0.05). The results demonstrated that treatment of the tumors in the nude mice with PC3 cell-derived exosomes carrying inhibited miR-183 impeded tumor growth.

The expression of miR-183 and TPM1 in tumor tissues of nude mice was examined by RT-qPCR assay. The results displayed that the expression of miR-183 was significantly decreased in tumor tissues of mice after treatment of exosomes carrying inhibition of miR-183 (*p* < 0.05) while the expression of TPM1 was significantly increased (Fig. 7c).

The TUNEL assay results revealed that the number of apoptosis in mice injected with exosomes derived from inhibitor-NC transfected-LNCaP cells was significantly increased (Fig. 7d), suggesting that exosomes with inhibited miR-183 promoted the apoptosis of tumor cells. In summary, inhibition of the exosomal miR-183 could inhibit the development of prostate tumors.

## Discussion

A significant society health care burden exists due to the high rate of prostate cancer morbidity and mortality, and fortunately, continued research has identified the involvement of miRNAs in the development and progression of prostate cancer [18]. Cancer-cells derived exosomes possess the capacity to transfer aberrantly expressed miRNAs and participate in process of invasion within tumor microenvironment [19]. Thus, the association between exosomal miRNA and occurrence of prostate cancer has increasingly become an area of significant research interest [9, 20]. More importantly, miR-183 has been highlighted to possess tumor-promoting properties in prostate cancer [21]. Hence, the current study was designed to explore the regulatory role of exosomal miR-183 in cell proliferation, migration, and invasion of prostate cancer. Previous well-established evidence has indicated the role of exosomal miR-183 as a contributor to cell proliferation and metastasis in prostate cancer with the involvement of TPM1.

Previous studies have been suggested that miR-183 is overexpressed in prostate cancer cells and plays crucial role in carcinogenesis [13, 17], which was largely consistent with our initial findings whereby miR-183 exhibited high levels of expression in prostate cancer cells. Accumulating evidence continues to highlight the ability of miR-183 to donate to malignant tumor progression and various metastatic processes. For example, the upregulation of miR-183-3p has been shown to facilitate lymph node metastasis in lung adenocarcinoma [22]. Besides, similar findings have been previously asserted highlighting the upregulation of miR-183-5p as an oncogenic element in non-small cell lung cancer [23]. Additionally, the downregulation of miR-182, a member of miR-183 family, inhibits invasion while promoting apoptosis in melanoma [24]. In an attempt to further elucidate the effect of miR-183 on prostate cancer cell proliferation, invasion and migration, gain- and loss-of-function experiments was conducted using mimics and inhibitors. The results obtained demonstrated that the overexpression of miR-183 promoted the proliferation, invasion and migration while inhibition of miR-183 suppressed proliferation, invasion and migration in cancer cells. Our data further emphasized the oncogenic role of miR-183 in prostate cancer. Crucially, a previous report concluded that miRNA-183 inhibits apoptosis and enhances proliferation in esophageal cancer by targeting PDCD4 [14]. In present study, it was predicted that miR-183 was able to target TPM1 gene which was further verified by the dual-luciferase reporter gene assay results. A key observation made during the current study indicated that TPM1 as a tumor suppressor gene was poorly expressed in prostate cancer cells. Emerging research has revealed that TPM1 may function as a tumor suppressor, highlighting its potential role in tumorigenesis as well as the development of a wide variety of malignancies. For instance, under-expressed TPM1 was identified in colorectal cancer and TPM1 exerts inhibitory role development of colorectal cancer cells [25]. TPM1 exhibits low levels of expression and has been reported to suppress cell proliferation and migration in addition to inducing the apoptosis of intrahepatic cholangiocarcinoma cells [26]. Moreover, TPM1 was conductive to restrain cell proliferation, angiogenesis and metastasis in renal cell carcinoma [27]. Collectively, the aforementioned findings in addition to the current study results highlight the inhibitory effect of TPM1 on tumorigenesis.

Accordingly, we considered the possibility that miR-183 may contribute to tumor growth and metastasis acceleration in prostate cancer via the downregulation of TPM1. Existing literature has suggested that miR-183 exerts pro-tumorigenic effects *via* targeting some tumor suppressor gene. For example, miR-183-5p has been reported to facilitate tumor metastasis and tumor growth in non-small cell lung cancer *via* downregulating PTEN [23]. Additionally, miR-183 may act to reduce the expression of the tumor suppressor gene AKAP12 in hepatocellular carcinomas [28]. Moreover, miR-183 has been further emphasized in previous investigations as an oncogenic factor that regulates 2 tumor suppressor genes including EGR1 and PTEN in synovial sarcoma, rhabdomyosarcoma, and colon cancer [29]. These findings were partially consistent with our results, whereby miR-183 was found to play a tumor-promoting role by targeting TPM1 in prostate cancer cells.

Moreover, our study results demonstrated that transfer of miR-183 by prostate cancer cells-derived exosomes promoted prostate cancer cell growth. Evidence has been previously presented suggesting that exosomal miRNA plays a key role in communication between cell and cell in cancer [30]. In our co-culture system, it was verified that miR-183 could be delivered into LNCaP cells by PC3 cells-derived exosomes. Furthermore, the transfer of miR-183 from PC3 cells-derived exosomes led to increase in proliferation, migration, and invasion in LNCaP cells. Consistent with our findings, exosomal miR-423-5p has been previously reported to enhance the sensitivity of breast cancer cells to DDP [31]. Also, exosomal miR-20a-5p has been demonstrated to accelerate bone metastasis of breast cancer [32]. At the same time, inhibition of exosomal miR-183 inhibited the proliferation, migration and invasion of prostate cancer cells. Additionally, we further confirmed that inhibition of exosomal miR-183 restrained tumor growth in nude mice.

## Conclusions

**Fig. 8:**
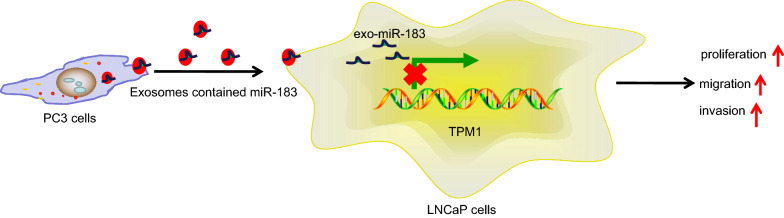
The mechanism graph of the regulatory function of exosomal miR-183 in prostate cancer. Exosomal miR-183 derived from PC3 cells facilitated prostate cancer LNCaP cell proliferation, migration, and invasion *via* downregulating TPM1

Taken together, the key findings of the present study collectively evidence that exosomal miR-183 derived from PC3 cells facilitates prostate cancer LNCaP cell proliferation, migration, and invasion *via* downregulating TPM1 (Fig. 8). Furthermore, the inhibition of exosomal miR-183 was found to impede cell proliferation, migration, and invasion in prostate cancer through the upregulation of TPM1. The results of our study highlight the oncogenic role of miR-183 in prostate cancer, presenting a potential target for novel therapy to curb the carcinogenesis in prostate cancer. However, the application of this strategy may face certain limitations including lack of effective exosome extraction techniques. Moreover, the optimal dose and number of injections of exosomes remain relatively unknown. Therefore, further investigations are required to identify such details.

## Data Availability

The datasets generated and/or analysed during the current study are available.

## Abbreviations

miRNAs: MicroRNAs
TPM1: Tropomyosin-1
GEO: Gene Expression Omnibus
FC: Fold change
3′UTR: 3′-Untranslated region
WT: Wild type
NC: Negative control
CCK-8: Cell counting kit-8
WST-8: Water-soluble tetrazolium salt-8
DLS: Dynamic light scattering
RT-qPCR: Reverse transcription-quantitative polymerase chain reaction
SDS-PAGE: Sodium dodecyl sulfate-polyacrylamide gel electrophoresis
PVDF: Polyvinylidene fluoride
RIPA: Radio-immunoprecipitation assay
TBS/T: Tris Buffered Saline with Tween
IgG: Immunoglobulin G
ECL: Enhanced chemiluminescence
SPF: Specific pathogen-free
TUNEL: Terminal deoxynucleotidyl transferase (TDT)-mediated dUTP-biotin nick end-labeling
H_2_O_2_: Hydrogen peroxide
ANOVA: Analysis of variance

## References

[1] Hayashi T, Fujita K, Matsushita M, Nonomura N (2019). Main Inflammatory Cells and Potentials of Anti-Inflammatory Agents in Prostate Cancer. Cancers (Basel).

[2] Birnbaum MD, Zhao N, Moorthy BT, Patel DM, Kryvenko ON, Heidman L, Kumar A, Morgan WM, Ban Y, Reis IM, Chen X, Gonzalgo ML, Jorda M, Burnstein KL, Zhang F (2019). Reduced Arginyltransferase 1 is a driver and a potential prognostic indicator of prostate cancer metastasis. Oncogene.

[3] Tang Y, Pan J, Huang S, Peng X, Zou X, Luo Y, Ren D, Zhang X, Li R, He P, Wa Q (2018). Downregulation of miR-133a-3p promotes prostate cancer bone metastasis via activating PI3K/AKT signaling. J Exp Clin Cancer Res.

[4] Doldi V, Pennati M, Forte B, Gandellini P, Zaffaroni N (2016). Dissecting the role of microRNAs in prostate cancer metastasis: implications for the design of novel therapeutic approaches. Cell Mol Life Sci.

[5] Stegeman S, Amankwah E, Klein K, O’Mara TA, Kim D, Lin HY, Permuth-Wey J, Sellers TA, Srinivasan S, Eeles R, Easton D, Kote-Jarai Z, Amin Al Olama A, Benlloch S, Muir K, Giles GG, Wiklund F, Gronberg H, Haiman CA, Schleutker J, Nordestgaard BG, Travis RC, Neal D, Pharoah P, Khaw KT, Stanford JL, Blot WJ, Thibodeau S, Maier C, Kibel AS, Cybulski C, Cannon-Albright L, Brenner H, Kaneva R, Teixeira MR, Consortium P, Cancer B, Spurdle AB, Clements JA, Park JY, Batra J (2015). A Large-Scale Analysis of Genetic Variants within Putative miRNA Binding Sites in Prostate Cancer. Cancer Discov.

[6] Nishikawa R, Goto Y, Sakamoto S, Chiyomaru T, Enokida H, Kojima S, Kinoshita T, Yamamoto N, Nakagawa M, Naya Y, Ichikawa T, Seki N (2014). Tumor-suppressive microRNA-218 inhibits cancer cell migration and invasion via targeting of LASP1 in prostate cancer. Cancer Sci.

[7] Bhagirath D, Yang TL, Bucay N, Sekhon K, Majid S, Shahryari V, Dahiya R, Tanaka Y, Saini S (2018). microRNA-1246 Is an Exosomal Biomarker for Aggressive Prostate Cancer. Cancer Res.

[8] Liu C, Xu X, Li B, Situ B, Pan W, Hu Y, An T, Yao S, Zheng L (2018). Single-Exosome-Counting Immunoassays for Cancer Diagnostics. Nano Lett.

[9] Huang X, Yuan T, Liang M, Du M, Xia S, Dittmar R, Wang D, See W, Costello BA, Quevedo F, Tan W, Nandy D, Bevan GH, Longenbach S, Sun Z, Lu Y, Wang T, Thibodeau SN, Boardman L, Kohli M, Wang L (2015). Exosomal miR-1290 and miR-375 as prognostic markers in castration-resistant prostate cancer. Eur Urol.

[10] Liu Q, Chen YQ (2010). A new mechanism in plant engineering: the potential roles of microRNAs in molecular breeding for crop improvement. Biotechnol Adv.

[11] Gam JJ, Babb J, Weiss R (2018). A mixed antagonistic/synergistic miRNA repression model enables accurate predictions of multi-input miRNA sensor activity. Nat Commun.

[12] Di Leva G, Garofalo M, Croce CM (2014). MicroRNAs in cancer. Annu Rev Pathol.

[13] Mihelich BL, Khramtsova EA, Arva N, Vaishnav A, Johnson DN, Giangreco AA, Martens-Uzunova E, Bagasra O, Kajdacsy-Balla A, Nonn L (2011). miR-183-96-182 cluster is overexpressed in prostate tissue and regulates zinc homeostasis in prostate cells. J Biol Chem.

[14] Yang M, Liu R, Li X, Liao J, Pu Y, Pan E, Yin L, Wang Y (2014). miRNA-183 suppresses apoptosis and promotes proliferation in esophageal cancer by targeting PDCD4. Mol Cells.

[15] Li J, Huang H, Sun L, Yang M, Pan C, Chen W, Wu D, Lin Z, Zeng C, Yao Y, Zhang P, Song E (2009). MiR-21 indicates poor prognosis in tongue squamous cell carcinomas as an apoptosis inhibitor. Clin Cancer Res.

[16] Kim R, Lee S, Lee J, Kim M, Kim WJ, Lee HW, Lee MY, Kim J, Chang W (2018). Exosomes derived from microRNA-584 transfected mesenchymal stem cells: novel alternative therapeutic vehicles for cancer therapy. BMB Rep.

[17] Dambal S, Baumann B, McCray T, Williams L, Richards Z, Deaton R, Prins GS, Nonn L (2017). The miR-183 family cluster alters zinc homeostasis in benign prostate cells, organoids and prostate cancer xenografts. Sci Rep.

[18] Zheng H, Bai L (2019). Hypoxia induced microRNA-301b-3p overexpression promotes proliferation, migration and invasion of prostate cancer cells by targeting LRP1B. Exp Mol Pathol.

[19] Milane L, Singh A, Mattheolabakis G, Suresh M, Amiji MM (2015). Exosome mediated communication within the tumor microenvironment. J Control Release.

[20] Ye Y, Li SL, Ma YY, Diao YJ, Yang L, Su MQ, Li Z, Ji Y, Wang J, Lei L, Fan WX, Li LX, Xu Y, Hao XK (2017). Exosomal miR-141-3p regulates osteoblast activity to promote the osteoblastic metastasis of prostate cancer. Oncotarget.

[21] Ueno K, Hirata H, Shahryari V, Deng G, Tanaka Y, Tabatabai ZL, Hinoda Y, Dahiya R (2013). microRNA-183 is an oncogene targeting Dkk-3 and SMAD4 in prostate cancer. Br J Cancer.

[22] Xu F, Zhang H, Su Y, Kong J, Yu H, Qian B (2014). Up-regulation of microRNA-183-3p is a potent prognostic marker for lung adenocarcinoma of female non-smokers. Clin Transl Oncol.

[23] Wang H, Ma Z, Liu X, Zhang C, Hu Y, Ding L, Qi P, Wang J, Lu S, Li Y (2019). MiR-183-5p is required for non-small cell lung cancer progression by repressing PTEN. Biomed Pharmacother.

[24] Segura MF, Hanniford D, Menendez S, Reavie L, Zou X, Alvarez-Diaz S, Zakrzewski J, Blochin E, Rose A, Bogunovic D, Polsky D, Wei J, Lee P, Belitskaya-Levy I, Bhardwaj N, Osman I, Hernando E (2009). Aberrant miR-182 expression promotes melanoma metastasis by repressing FOXO3 and microphthalmia-associated transcription factor. Proc Natl Acad Sci USA.

[25] Mlakar V, Berginc G, Volavsek M, Stor Z, Rems M, Glavac D (2009). Presence of activating KRAS mutations correlates significantly with expression of tumour suppressor genes DCN and TPM1 in colorectal cancer. BMC Cancer.

[26] Yang W, Wang X, Zheng W, Li K, Liu H, Sun Y (2013). Genetic and epigenetic alterations are involved in the regulation of TPM1 in cholangiocarcinoma. Int J Oncol.

[27] Narbonne JF, Suteau PM, Grolier PA, Daubeze MC (1988). Metabolic effects of a polychlorinated biphenyl (Phenoclor DP6) on mullets, Chelon labrosus. Bull Environ Contam Toxicol.

[28] Goeppert B, Schmezer P, Dutruel C, Oakes C, Renner M, Breinig M, Warth A, Vogel MN, Mittelbronn M, Mehrabi A, Gdynia G, Penzel R, Longerich T, Breuhahn K, Popanda O, Plass C, Schirmacher P, Kern MA (2010). Down-regulation of tumor suppressor A kinase anchor protein 12 in human hepatocarcinogenesis by epigenetic mechanisms. Hepatology.

[29] Sarver AL, Li L, Subramanian S (2010). MicroRNA miR-183 functions as an oncogene by targeting the transcription factor EGR1 and promoting tumor cell migration. Cancer Res.

[30] Falcone G, Felsani A, D’Agnano I (2015). Signaling by exosomal microRNAs in cancer. J Exp Clin Cancer Res.

[31] Wang B, Zhang Y, Ye M, Wu J, Ma L, Chen H (2019). Cisplatin-resistant MDA-MB-231 cell-derived exosomes increase the resistance of recipient cells in an exosomal miR-423-5p-dependent manner. Curr Drug Metab.

[32] Guo L, Zhu Y, Li L, Zhou S, Yin G, Yu G, Cui H (2019). Breast cancer cell-derived exosomal miR-20a-5p promotes the proliferation and differentiation of osteoclasts by targeting SRCIN1. Cancer Med.

